# Gastrointestinal parasitic infections: Prevalence and risk factors in West Ismailia, Arab Republic of Egypt

**DOI:** 10.1186/s13099-024-00622-y

**Published:** 2024-06-19

**Authors:** Shahira Abdelaziz Ali Ahmed, Samar Farag Mohamed, Heba Sayed El-Mahallawy, Annalisa Quattrocchi, Panagiotis Karanis

**Affiliations:** 1 Department of Parasitology, Faculty of Medicine, Suez Canal University, Ismailia, 41522 Egypt; 2 Department of Family Medicine, Faculty of Medicine, Suez Canal University, Ismailia, 41522 Egypt; 3 Department of Zoonoses, Faculty of Veterinary Medicine, Suez Canal University, Ismailia, 41522 Egypt; 4 Department of Primary Care and Population Health, University of Nicosia Medical School, Nicosia, 24005, CY-1700 Cyprus; 5 Department of Basic and Clinical Sciences, University of Nicosia Medical School, Nicosia, 24005, CY- 1700 Cyprus

**Keywords:** Gastrointestinal parasites, Human, Prevalence, West Ismailia, Risk factors, Egypt

## Abstract

**Background:**

This study aimed to determine the prevalence of gastrointestinal parasites (GIP) in the rural community of West Ismailia and its associated risk factors. Human infection by GIP is natural and expected. There are few records concerning parasitic infection in the rural areas of the Ismailia Governorate.

**Methods:**

From 520 individuals, sociodemographic and risk factors information were retrieved. Fecal samples were collected, concentrated, and tested for GIP infection using a microscopic examination.

**Results:**

The West Ismailia study population had a 40.4% prevalence of GIP infection, including single and concomitant parasite infections. The most common cause of GIP infection was protists (38%). *Entamoeba* sp., *Blastocystis* sp., and *G. duodenalis* were the most common parasites. Poly-parasitism was prevalent within the West Ismailia region. Age, abdominal symptoms, perianal itching, ownership of numerous animal species, exposure to turbid water, previous parasitic infection (PPI), and non-treatment reception of PPI were all considered significant factors associated with GIP infection. Specific individuals from the same family have been observed to have identical GIP.

**Conclusion:**

GIP infection remains underestimated in rural areas. Periodic screening and treatment for GIP infection in children and public education on GIP hazards and prevention, focusing on personal hygiene, are recommended. Family members of affected individuals should be screened and treated for GIP.

**Supplementary Information:**

The online version contains supplementary material available at 10.1186/s13099-024-00622-y.

## Background

Gastrointestinal parasites (GIP) can colonize the gastrointestinal tracts of humans and animals. The most typical way for these parasites to spread via the fecal-oral pathway is through ingesting contaminated food, infected meat, water, soil, or fomites. Direct transmission via person-to-person or animal-to-person is also possible [1–3].

Gastrointestinal parasites are strongly associated with poverty, a lack of or inadequate access to safe potable water, poor sanitation, poor hygiene practices, and low levels of education. Although they disproportionately affect humans in low- and middle-income countries (LMICs), these infections contribute significantly to the disease burden in wealthy nations [2, 4–7].

It is estimated that about two billion people worldwide are infected with GIP, which is well-documented to increase morbidity and mortality [3, 6, 8, 9]. The predominant symptom of GIP infections is diarrhea, which is more prevalent in LMICs. According to GBD Diarrheal Diseases Collaborators (2017), diarrheal diseases are the leading cause of mortality and disability-adjusted life years (DALYs) among individuals of all ages worldwide [10]. They were responsible for the fatalities of 1.6 million individuals, while one-third of these deaths were in children under the age of five, primarily caused by pathogens, such as viruses, bacteria, and protists [7]. Millions of children are highly susceptible to infection with protists and helminthic parasites. At the same time, GIP infection was associated with malabsorption, weight loss, anemia, stunting, learning difficulties, mental retardation, and intellectual problems [1, 7, 11, 12].

African countries are the most poverty-stricken regions of the world [13]. African preschool and school-aged children continue to be a significant GIP burden population. Poor environmental sanitation and socioeconomic status, insufficient safe water, low or no maternal education, and poor hygienic practices (close contact with soil, eating without washing hands, purchasing contaminated food from the school canteen, walking barefoot, and not trimming fingernails) plague schoolchildren in Africa [1, 12, 14–18]. Therefore, GIP mass drug administration (MDA) strategies have been extensively applied in African countries in the past five years [1, 19–23]. However, risky communities cannot avert GIP reinfections even if the disease has been successfully treated [24].

The Arab Republic of Egypt (ARE) ranked third among the included reports of GIP, after Ethiopia and Nigeria. This highlights the urgent need for continued research and intervention, as nearly all GIP infections were reported with varied pooled prevalence. ARE depends on the River Nile, a surface water source contaminated by human, industrial, and agricultural runoff [25]. Sewage and industrial effluents may be released into the River Nile with limited treatment in many Egyptian rural communities [26]. Consequently, Nile water contamination is a significant source of GIP [27]. Water, sanitation, and hygiene (WASH) have been prioritized in endemic settings to supplement MDA programs [28]. With the assistance of WASH, molluscicide, and MDA programs, ARE was able to reduce the quantity of *Schistosoma* intermediate hosts (snails), and its prevalence decreased from 10 to 30% to > 3% in 2010 [29].

One of the east-west tributaries of the River Nile, formerly known as the Sweet Water Canal, is located in the Ismailia governorate [30]. Sweet Water Canal was recently reported as a possible source of GIP, posing a high risk to the rural population in Ismailia [18]. However, the prevalence of GIP and risk factors in such governorates needed to be better identified.

Different governorates in ARE discussed the prevalence and risk factors of GIP, which appeared to vary by location [31–35] and required prevalence rate evaluation [36]. The current study aims to investigate the prevalence of GIP and its associated factors in the West Ismailia region.

## Methods

### Study area and study population

This study used a cross-sectional design in the Ismailia governorate in north-eastern ARE. The government of Ismailia is a well-known metropolis on the banks of the Suez Canal, bordered to the north by Port Said and Suez to the south [37]. Ismailia is located between 30° 35’ 47.3712’’ N and 32° 16’ 17.2524’’ E, at 10.265 m above mean sea level [38], and it had an estimated population of about 1,442,402 in 2023 [39].

**Fig. 1:**
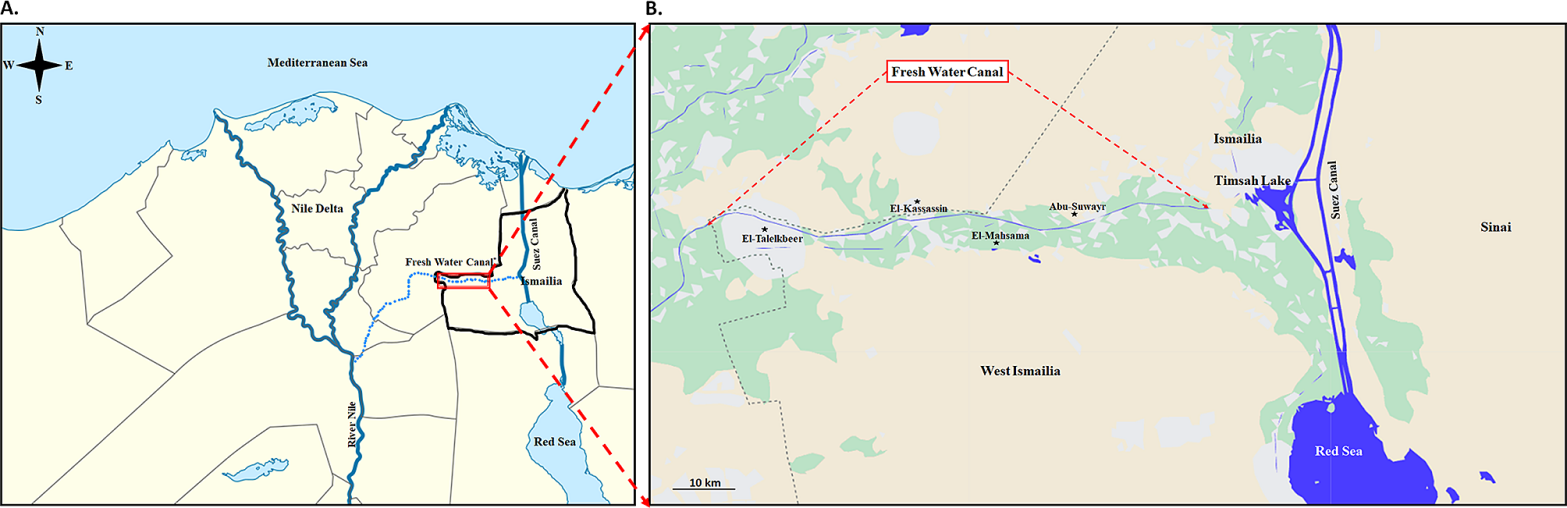
(**A**) The Arab Republic of Egypt Nile Delta map. Geographic limits of the Nile Delta are N: 31°55’ N; S: 29°22’ N; W: 28°52’ E; O: 33°4’ E. Red rectangle refers to the study area; The red rectangle refers to the study area; the blue dot line identifies Ismailia Fresh Water Canal as a branch of the River Nile. ^*^Fresh Water Canal = Sweet Water Canal = Ismailia Canal. (**B**) A magnified map depicts the current study localities (black stars) dispersed throughout West Ismailia. El-Mahsama Family Practice Center is located in El-Mahsama locality

West Ismailia is a rural municipal division of Ismailia that encompasses the localities of “El-Kassassin, El-Mahsama, El-Talelkbeer, and Abu-Suwayr”, which are situated along the Sweet Water Canal geographical line (Fig. 1) [18]. This population’s livelihood is predominately agricultural, with limited access to purified water and sanitary facilities.

The Sweet Water Canal, also known as Fresh Water Canal and currently known as Ismailia Canal, is one of the most essential branches of the River Nile and serves as the primary water source for West Ismailia. The canal traverses the Ismailia governorate from east to west, from Lake Timsah to Suez and Port Said, supplying the arid region with Freshwater. The canal expanded agricultural communities along its sides [39], and continues to be West Ismailia’s primary source of drinking and irrigation water [40, 41] (Fig. 1).

Participants in the four rural localities of West Ismailia were recruited using a convenient sampling strategy. The sampling was conducted in the nearest sampling area, and participants were approached at their homes.

To be eligible for involvement in the study, participants had to reside at the location for at least one month before their commencement.

Individuals who were unable to provide informed consent on account of a severe mental illness or cognitive impairment were excluded from the study. Epi Info TM Stat Calc version 7.2.4.0 (Centers for Disease Control and Prevention, Atlanta, GA, USA) was utilized to determine the sample size of 459. The calculation was predicated on the lowest prevalence value of GIP infection derived from Elmonir et al. (2021), with a 5% margin of error and a 95% confidence level. An additional 20% was added to the calculated sample size to consider refusals, totaling 520 samples.

Due to ethical considerations regarding the residents’ requests, the current study incorporated seven unintended participants from the “urban area” of Ismailia city. The seven Ismailia residents were visiting their relatives in the West Ismailia region.

### Questionnaire survey

Team members of El-Mahsama Family Practice Center (FPC) informed the population about the study, assisted by Arabic-speaking health center representatives and a community employee. All participants were interviewed face-to-face to obtain sociodemographic and risk factor information. The parents, guardians, or family heads who signed the informed permission form were interviewed for participants under eighteen and senior individuals in someone else’s care.

The questionnaire captured the following information:

- Demographic characteristics (name, age, sex, residence, phone number).
- Zoonotic exposure characteristics (owing animals, type of animals, direct contact with animals, type of animal farm ground).
- Water facilities characteristics (access to potable water, description of water, type of water supply to human consumption, type of water source for animals).
- Gastrointestinal (GIT) symptoms (including diarrhea, abdominal pain, bloody stool, vomiting, fever, dehydration, and perianal itching).
- History of parasitic infection, including received treatment.

### Samples collection and parasitological examination

Each participant had a clean, labeled plastic container with an applicator stick. Participants were verbally informed in Arabic of the collection and transfer instructions of the required stool samples. Fecal samples were collected from the adjacent sampling area regardless of age or gender. Occasionally, the entire household was included in the sampling.

All samples were immediately labeled with a participant-specific identifier consisting of a code number in order of collection. Stool samples were transported in an ice box to the Parasitology Laboratory at Suez Canal University for parasitological examination.

The volume of fecal samples (a full tablespoon, or 15–20 g) served as a guide for rolling in the fecal samples received. Each sample was thoroughly mixed, after which a slide was immediately prepared for wet mounting in saline and Lugol’s iodine solution for light microscopy examination.

The fecal-saline suspension was then sieved through a four-layer gauze and concentrated using formalin ethyl acetate.

Negative results are documented when no parasites are detected, and positive results are recorded when single or multiple parasitic infections are detected. In cases of polyparasitism, the number of parasites was recorded. Two experienced laboratory microscopists with at least three years of work experience examined each sample. Anonymously and by the standard operating procedure for examinations, another laboratory microscopist conducted a cross-check of the slides.

### Ethical consideration

The study protocol and questionnaire were reviewed and approved by Suez Canal University’s Research and Ethics Review Committee (Approval number: 5424). All survey participants received free medical consultations, appropriate treatment according to El-Mahsama FPC guidelines, and referral to competent specialists as needed. Participants and their families infected with parasites received health education to avoid transmission routes and reinfection.

### Definitions

Gastrointestinal parasitic infection is divided into “Mono/single infection” (i.e. presence of one parasitic infection and no other parasites in the fecal sample); “Double infection” (i.e. presence of two different parasitic infections in the fecal sample); “Triple infection” (i.e. presence of three various parasitic infections in the fecal sample); “Quadruple infection” (i.e. presence of four different parasitic infections in the fecal sample); “Concomitant parasitic infection” (i.e. infection with multiple parasitic species either protists with protists, protists with helminths, or helminths with helminths); “Previous parasitic infection (PPI)” (i.e. participants who have been infected with parasites within the past year).

Helminths refer to:

- *Enterobius vermicularis* (*E. vermicularis*).
- *Hymenolepis nana* (*H. nana*).
- *Strongyloides stercoralis* (*S. stercoralis*).
- *Diphyllobothrium latum* (*D. latum*).
- *Taenia* sp.

Protists refer to:

- *Giardia duodenalis (G. duodenalis)*.
- *Entamoeba histolytica/dispar/moshkovskii* *(E. histolytica complex).*
- *Cyclospora cayetanensis* (*C. caytanensis*).
- *Entamoeba coli* (*E. coli*).
- *Chilomastix mesnili* (*C. mesnili*).
- *Blastocystis* sp.

### Statistical analysis

Descriptive statistics are presented as frequencies for categorical variables or mean and standard deviation (SD) for continuous variables.

To identify factors associated with having a GIP infection (outcome), univariable logistic regression analysis was performed, and the crude odds ratios (cOR) and 95% confidence intervals (95% CI) were calculated. A similar approach was conducted for the three most prevalent parasites causing GIP infection (*Blastocystis* sp., *G. duodenalis*, and *E. coli*).

Variables with a *p*-value < 0.2 were retained for multivariable analysis. Backward stepwise selection method was used for multivariable logistic regression. The adjusted odds ratios (aOR) and their 95% CIs were calculated.

A *p*-value < 0.05 was considered statistically significant. All analyses were performed using Stata software, version 17 (StataCorp, College Station, TX, US).

## Results

### Characteristics of West Ismailia population

The West Ismailia sample of 520 individuals comprised nearly equal numbers of both genders, with the majority (60%) under 15 years of age and residing in the El-Mahsama region (Table 1). Domesticated animals were reported by 91% of the participants; pets and poultry constituted the majority of animal ownership. Approximately 76.4% of participants had direct contact with their animals, and most of their animal farms were constructed with sand (Additional File 1, Table S1).

**Table 1:** Sociodemographic characteristics of West Ismailia participants (520 individuals)

Variable	Category	Number	%
Gender	Female	303	58.3
	Male	217	41.7
Age group (years)^a^	0–4	71	13.7
	5–9	129	24.8
	10–14	111	21.4
	15–34	120	23.1
	35+	89	17.1
Residence^b^	Abu-Suwayr	44	8.5
	El-Kassassin	108	20.8
	El-Mahsama	225	43.3
	El-Talelkbeer	136	26.2
	Ismailia^c^	7	1.4

^a^ Age mean (SD) = 18.2 (16.3); ^b^ All locations are rural areas within the West Ismailia municipality, except for Ismailia, an urban area unrelated to West Ismailia; ^c^ The seven Ismailia residents were visiting their relatives in the West Ismailia region

Most of West Ismailia participants had access to potable water; however, approximately 40% reported consuming turbid or yellow water. The predominant water source accessible was tap water, which constituted 71% for human consumption and 70% for animal use (Additional File 2, Table S2). GIT symptoms were reported in 71% of the West Ismailia sample, with abdominal discomfort being the most frequently reported (61.5%) (Additional File 3, Table S3). A PPI was reported by nearly half; however, treatment for their GIP infection was only administered to 39% of this subset. The species of *Entamoeba* were most frequently referenced by the study population, followed by *E. vermicularis* (Additional File 4, Table S4).

### Prevalence of GIP infection in the West Ismailia population

The prevalence of GIP infection among the study population of West Ismailia was 40.4%, comprised of both single and concomitant parasitic infections. The predominant cause of GIP infection was protists (38%), while helminths’ prevalence was 2.5%. The most frequent parasitic infections were the species of *Entamoeba*, *Blastocystis*, and *G. duodenalis* (Table 2).

**Table 2:** Prevalence of gastrointestinal parasites infection in West Ismailia population (520 individuals)

Variable	Category	Number	Prevalence %
Prevalence of GIP		210	40.4
Number of GIP infection	Single	161	31.0
	Double	38	7.3
	Triple	10	1.9
	Quadruple	1	0.2
Prevalence of protists		197	37.9
	*Blastocystis* sp.	80	15.4
	*Giardia duodenalis*	64	12.3
	*Entamoeba coli*	62	11.9
	*Entamoeba* complex*	30	5.8
	*Cyclospora cayetanensis*	16	3.1
	*Chilomastix mesnili*	6	1.2
Prevalence of helminths		13	2.5
	*Hymenolepis nana*	6	1.2
	*Enterobius vermicularis*	4	0.8
	*Diphyllobothrium latum*	1	0.2
	*Strongyloides stercoralis*	1	0.2
	*Taenia* sp.	1	0.2

GIP: Gastrointestinal parasites; *Entamoeba complex: *Entamoeba histolytica*/*dispar*/*moshkovskii*

**Fig. 2:**
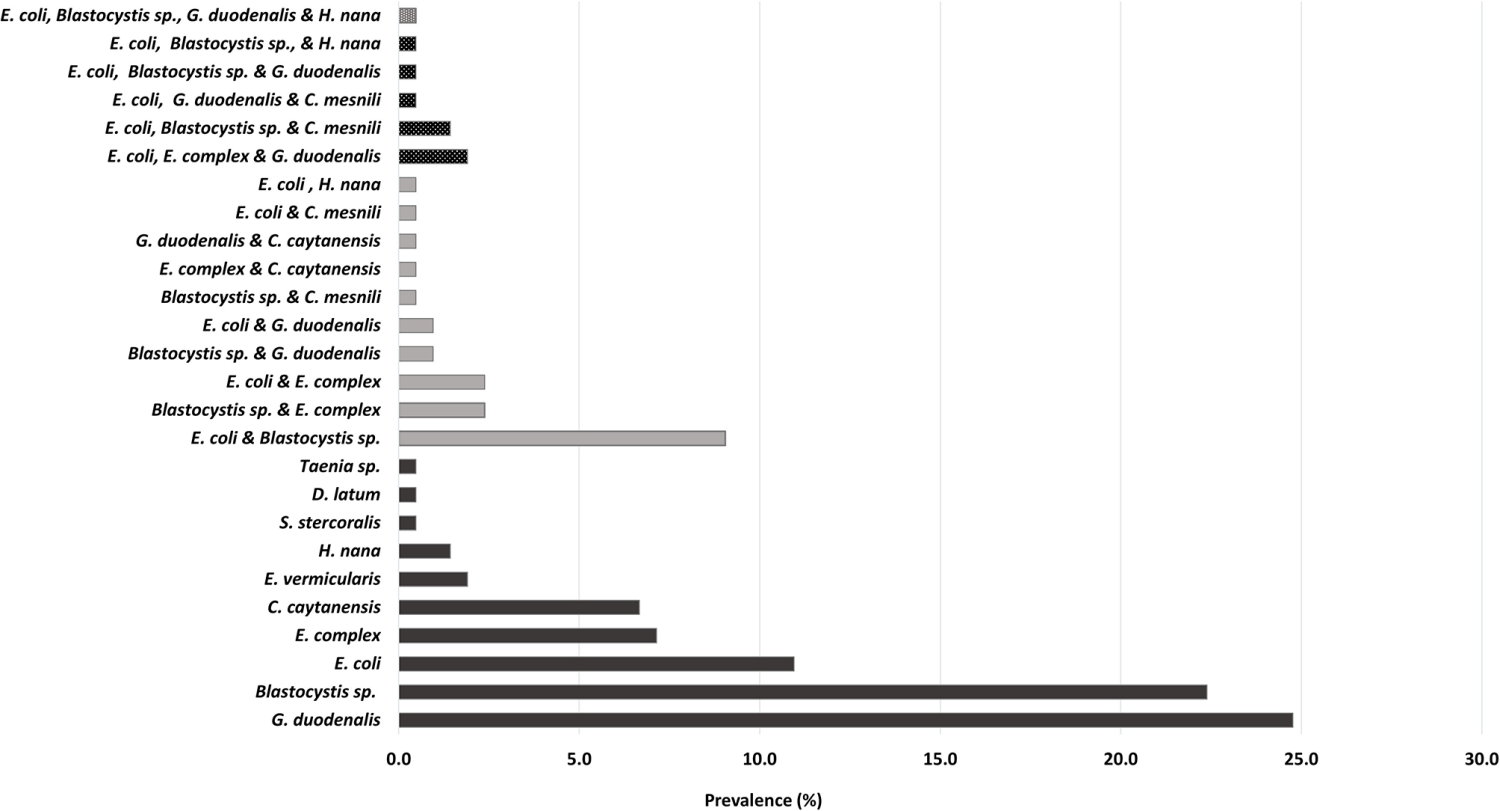
Distribution of mono- and concomitant-parasitic infection in West Ismailia populations. *G. duodenalis*: *Giardia duodenalis*; *E. coli*: *Entamoeba coli*; *E.* complex: *Entamoeba histolytica*/*dispar*/*moshkovskii*; *C. caytanensis*: *Cyclospora cayetanensis*; *E. vermicularis*: *Enterobius vermicularis*; *H. nana*: *Hymenolepis nana*; *S. stercoralis*: *Strongyloides stercoralis*; *D. latum*: *Diphyllobothrium latum*; *C. mesnili*: *Chilomastix mesnili*

Out of 520 participants, 161 individuals (31%) were found to be mono-infected with GIP (Table 2). The most frequently reported parasites in mono-infection cases were *G. duodenalis*, found in 52 cases (24.8%), *Blastocystis* sp. in 47 (22.4%), and *Entamoeba* sp. in 38 (18.1%). The double parasitic infection involving *E. coli* and *Blastocystis* sp. occurred most frequently in 19 cases (9%). The most prevalent parasite in the West Ismailia population, either in singleton or concurrent infections, was *Blastocystis* sp. (Fig. 2).

### Factors associated with having a GIP infection

According to univariable logistic regression analysis, residing in El-Mahsama, presence of any symptoms, abdominal symptoms and perianal itching, number of animal species owned, utilization of turbid water, PPI, and lack of treatment receipt for PPI were all significant characteristics associated with higher odds of GIP infection, while increasing age, possession of a pump for water supply and buying water containers showed a protective effect. (Table 3). The variables that remained significant in multivariable analysis were age (per 10-year increase aOR = 0.83, 95% CI: 0.72–0.96), having abdominal symptoms (aOR = 5.46, 95% CI: 3.08–9.69), having perianal itching (aOR = 3.79, 95% CI: 1.57–9.15), number of animal species owned (per one species increase aOR = 1.42, 95% CI: 1.03–1.97), having turbid water as opposed to clear water (aOR = 2.53, 95% CI: 1.44–4.47), PPI (aOR = 2.03, 95% CI: 1.14–3.62), and PPI non-treatment receipt (aOR = 2.11, 95% CI: 1.11–4.01).

**Table 3:** Risk factors associated with gastrointestinal parasitic infection in West Ismailia population (univariable and multivariable analysis)

Factor	Category	GIP Infection					
		cOR	*p*-value	95% CI	aOR	*p*-value	95% CI
Gender^a^	Male	1.12	0.542	0.78–1.59	-		
Age	(per 10-year increase)	0.82	0.001*	0.73–0.93	0.83	0.014*	0.72–0.96
Residence^b^	Abu-Swayer	1.92	0.072	0.94–3.92	-		
	El-Kassassin	1.70	0.056	0.99–2.93			
	El-Mahsama	2.85	< 0.001*	1.80–4.53			
	Ismailia	0.46	0.483	0.05–3.98			
Symptoms^c^	Any	4.78	< 0.001*	2.99–7.64	5.46	< 0.001*	3.08–9.69
	Diarrhea	1.30	0.183	0.89–1.90	-		
	Blood in stools	1.49	0.534	0.43–5.20	-		
	Vomiting	0.69	0.249	0.37–1.29	-		
	Fever	0.94	0.816	0.54–1.63	-		
	Abdominal pain	3.52	< 0.001*	2.37–5.23	-		
	Dehydration	0.86	0.749	0.33–2.21	-		
	Perianal itching	4.11	< 0.001*	1.86–9.08	3.79	0.003*	1.57–9.15
Zoonotic exposure	Pet^c^	1.71	0.004*	1.18–2.48	-		
	Livestock^c^	1.27	0.230	0.86–1.87	-		
	Poultry^c^	1.64	0.066	0-97-2.77	-		
	No. of animal’s species (continuous)	1.39	0.003*	1.12–1.72	1.42	0.035*	1.03–1.97
	Direct contact with animals^c^	0.90	0.627	0.59–1.38	-		
Water description^d^	Turbid	2.25	< 0.001*	1.51–3.37	2.53	0.001*	1.44–4.47
	Yellow	0.66	0.221	0.34–1.29	0.46	0.073	0.20–1.07
Water supply to human	Buying containers	0.42	0.001*	0.25–0.71	-		
	Pump	0.34	0.016*	0.14–0.82			
	Tank	1.18	0.843	0.23–5.91			
	Tap with filter	0.43	0.066	0.18–1.06			
Water source to animal^e^	Canal	0.54	0.169	0.23–1.30	0.67	0.497	0.21–2.12
	Ground water	1.89	0.316	0.54–6.59	1.81	0.411	0.44–7.39
	Pump	0.32	< 0.001*	0.19–0.52	0.47	0.015*	0.25–0.86
PPI	PPI^c^	4.55	< 0.001*	3.11–6.65	2.03	0.016*	1.14–3.62
	TTT of PPI^f^	3.01	< 0.001*	1.81–5.03	2.11	0.023*	1.11–4.01

Superscripts are variables that have a reference category; ^a^ Female; ^b^ El-Talelkbeer; ^c^ Yes; ^d^ Clear; ^e^ Tap; ^f^ No; *p-value < 0.05; GIP: Gastrointestinal parasites; cOR: Crude odds ratio; aOR: Adjusted odds ratio; CI: Confidence interval; PPI: Previous parasitic infection; TTT: Treatment

### Factors associated with the three most common GIP (***Entamoeba ***sp., ***Blastocystis*** sp., ***G. duodenalis***)

In univariable logistic regression analysis of *Entamoeba* sp. infection (*E. coli* and *E. histolytica* complex), the area of residence, any symptoms, abdominal symptoms, and PPI were significantly and positively associated with GIP infection. Multivariable logistic regression confirmed that those reporting at least one symptom, reporting perianal itching, having direct contact with animals, and having a PPI were two times more likely to be positive for *Entamoeba* sp. infection than the counterparts after adjusting for confounding (Additional File 5, Table S5).

Residing in El-Mahsama, having at least one symptom, as well as having abdominal pain, having animals (pet or poultry or more than one type), having turbid water, having PPI, and not being treated for it were significantly associated with *Blastocystis* sp. infection. Multivariable regression confirmed the association with having PPI (aOR: 24.56; 95% CI: 4.81-125.32) and residing in El-Mahsama (aOR: 3.05; 95% CI: 1.00-9.27) (Additional File 6, Table S6).

Risk factors for *G. duodenalis* infection were age, having any type of symptomatology, reporting perianal itching, having livestock animals, having more than one type of animal, consumption of tank water, utilizing ground water for animal, and having PPI. After adjusting for confounding at multivariable analysis, the most substantial factors for *G. duodenalis* infection were the type of water supply to humans: consuming tank water (aOR: 632.47; 95% CI: 13.62-29377.50) or from purchased containers (aOR: 12.06; 95% CI: 1.20-121.51), compared to tap water with the filter. In addition, the association with age was also confirmed, showing a clear inverse trend (the likelihood of infection increases as age decreases). Furthermore, having livestock animals and more than one type of animal and having any symptoms remained positively associated (Additional File 7, Table S7).

### PPI and their relation to the current infection and anti-parasitic therapy receipt

*Entamoeba* sp. was identified in 38 participants out of 265 individuals with PPI (Additional File, Table S4); of these, 12 (33%) reported a current infection (present infestation with any type of parasite), and 4 (33%) had a present GIP infection caused by *Entamoeba* sp. Fifteen participants, all of whom had a current GIP infection, reported *H. nana* as the PPI; however, only one (6.7%) had *H. nana* as the causative present parasite. PPI was reported for *E. vermicularis*, *Taenia sp.*, and *A. lumbricoides*; however, none of these reports documented the parasite as identical to the present one (Table 4).

**Table 4:** Previous parasitic infections (PPI) and their relation to the current infection

Parasite	Total PPI	Current infection (with any type of parasite)	Current infection (with the same parasite as PPI)
Entamoeba*	38	12	4
*H. nana*	15	15	1
*E. vermicularis*	25	13	0
*Taenia* sp.	2	0	0
*A. lumbricoides*	1	0	0

*Entamoeba coli, *Entamoeba histolytica*/*dispar*/*moshkovskii*

A significant proportion of the study population (approximately 63%) with PPI infection did not receive anti-parasitic treatment for their GIP infection. Specifically, those with unknown GIP infection, amoebiasis, schistosomiasis, hymenolepiasis and enterobiasis were least likely to take their medications or adhere to their prescribed treatment regimens (Table 5).

**Table 5:** Previous parasitic infection and its relationship to anti-parasitic therapy

PPI	Treatment receipt		*p*-value
	No (number = 163)	Yes (number = 103)	
Entamoeba*	16 (9.8%)	22 (21.4%)	< 0.001
*A. lumbricoides*	0 (0%)	1 (1%)	
*E. vermicularis*	6 (3.7%)	19 (18.5%)	
*H. nana*	7 (4.3%)	8 (7.8%)	
*Schistosoma* sp.	9 (5.5%)	3 (2.9%)	
*Taenia* sp.	0 (0%)	2 (1.9%)	
Yes, but unknown GIP infection	125 (76.7%)	48 (46.6%)	

*Entamoeba coli, *Entamoeba histolytica*/*dispar*/*moshkovskii*

### Members of the same household and the GIP they share

Two hundred and ninety-four (56.5%) of the study individuals were members of the same household, making up 100 households. The number of household members varied from 2 to 9 per household. GIP was not detected in 31 households, while in 51 households, family members differed in the presence/absence of GIP infection. In eighteen households, at least two members within the same household were diagnosed with the same GIP infection (Table 6).

**Table 6:** Distribution of gastrointestinal parasites infection among family members residing in the same household

Family No.	Total number of household members	Members infected with identical parasite	GIP infection within the same dwelling			
			*Blastocystis* sp.	*G. duodenalis*	*Entamoeba* sp.	*C. cayetanensis*
F1	3	3	3	-	-	-
F2	6	2	-	2	-	-
F3	4	2	-	2	-	-
F4	2	2	-	-	2 (*E. coli*)	-
F5	3	3	-	-	3 (*E. histolytica* complex)	-
F6	2	2	-	2	-	-
F7	2	2	2	-	-	-
F8	9	3	-	3	-	-
F9	3	2	-	-	-	2
F10	3	2	-	2	-	-
F11	3	2	2	-	-	-
F12	6	2	-	-	2 (*E. coli*)	-
F13	3	2	-	2	-	-
F14	3	2	-	-	2 (*E. coli*)	-
F15	2	2	-	-	2 (*E. coli*)	-
F16	6	4 (2)*	4	-	2 (*E. coli*)	-
F17	2	2	-	2	-	-
F18	2	2	-	-	-	2

GIP: Gastrointestinal parasites; *G. duodenalis*: *Giardia duodenalis*; *E. coli*: *Entamoeba coli*; *E. histolytica* complex: *Entamoeba histolytica*/*dispar*/*moshkovskii*; *C. caytanensis*: *Cyclospora cayetanensis*. *Two members with a *Blastocystis* infection also have an *E. coli* co-infection

The prevalence of GIP infection among individuals residing in the same domicile was 37.8%. However, GIP prevalence among individuals who did not have other family members in the study was 43.8%. This distinction was not statistically significant (*p* = 0.164).

### Pinworm infection between history of perianal itching and microscopic diagnosis

**Fig. 3:**
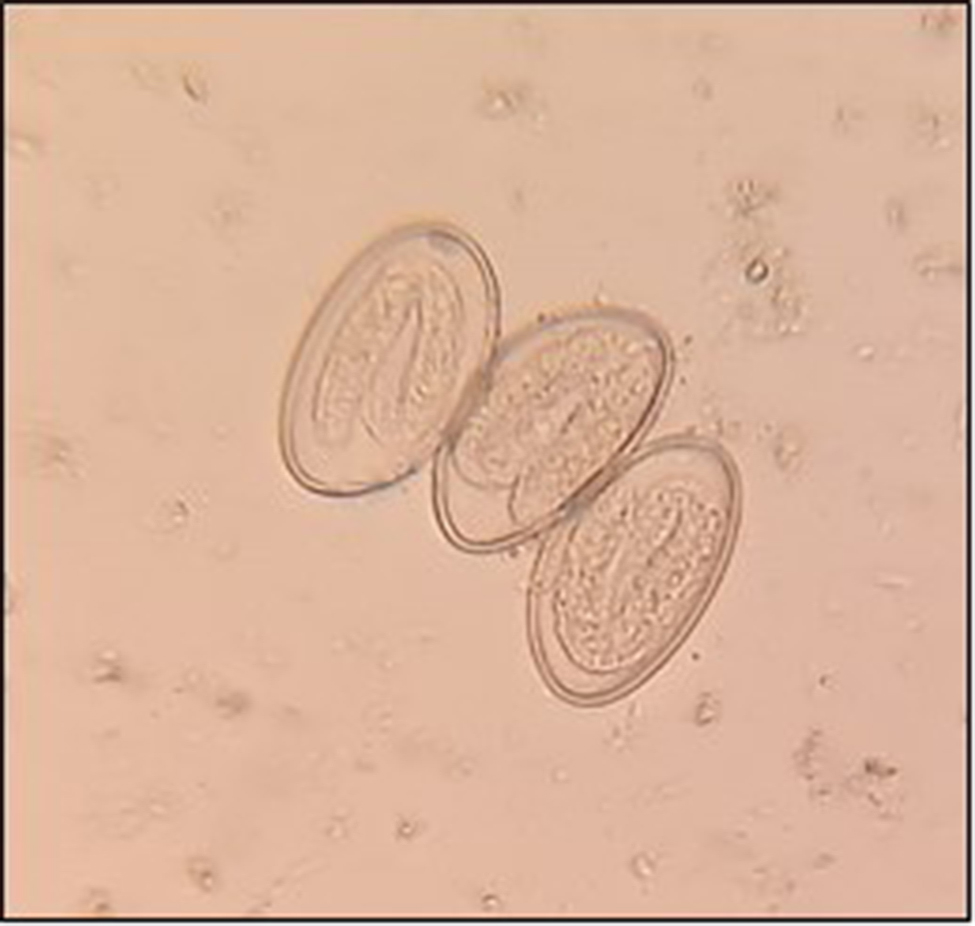
Eggs of *Enterobius vermicularis* in the stool of a heavily infected patient. 400x magnification

Thirty-two individuals reported having perianal itching, and of these, 13 (40.6%) reported having *E. vermicularis* as their PPI (OR: 27.1; *p* < 0.001). In addition, each of the four participants (12.5% of the total) who currently have *E. vermicularis* has previously reported having perianal itching. Heavy infection ranging from three to five eggs per field was found in each of the four people who tested microscopically positive for *E. vermicularis* (Fig. 3).

## Discussion

The current study investigates the prevalence of GIP and the associated risk factors in West Ismailia, ARE. Conducting continuous epidemiological surveys to ascertain the prevalence of GIP infections across various communities is crucial for identifying high-risk areas and formulating effective preventive and control strategies [31].

Forty percent of the 520 individuals examined in this study were infected with at least one parasite. The observed prevalence rate was comparatively lower than those previously documented in other regions of ARE, including Gharbia governorate (46.2%) [31], Zagazig district (56%) [42], and Cairo (59.6%) [32]. Nevertheless, it surpassed the prevalence among school students in Aswan (31%) [33]. The prevalence of GIP infection exhibits significant variability across different regions of the world, with the highest rates observed in Argentina (92.7%) [43], Ecuador (63.2%) [44], and Duhok, Iraq (62.26%) [45] and the lowest rates observed in Algeria (33.3%) [46]. Environmental factors, cultural differences, identification methods, and variations in personal sanitation practices among the populations under study may account for these differences.

In contrast to protists infection (37.9%), the prevalence of helminths was comparatively low at 2.5%. This finding was consistent with previous GIP prevalence studies conducted in ARE [32, 33, 42] and other regions [43]. The reduction in the prevalence of intestinal helminths could be attributed to the implementation of anti-helminthic community-based control programs [47]. Similarly, research conducted in Gabon has documented a decline in the incidence of intestinal helminths after the implementation of anti-helminthics [48]. On certain occasions, individuals residing in West Ismailia rural areas might also procure anti-helminth treatment and apply it to their families without initially seeking medical advice.

In this investigation, *Blastocystis* sp. emerged as the most commonly encountered protists, followed by *G. duodenalis* and *Entamoeba* sp. Earlier researchers reported similar results in various Egyptian governorates [31, 32, 42]. The same circumstances were observed in Algeria, Ecuador, Kenya, and Morrocco [44, 46, 49, 50]. These parasitic protists share common potential transmission sources, including human-to-human, zoonotic, waterborne, and foodborne concerning poor hygienic practices and poverty [1, 5]. *Blastocystis* sp. was identified as one of the most prevalent protists in African nations, with the highest estimated prevalence range exceeding 50% [1]. However, it is not listed among parasites in parasitological examination reports in ARE.

Additionally, *G. duodenalis* was the most predominant parasite documented across African countries and was associated with severe to moderate diarrhea in healthy adults [1]. In the third rank of protist infection of the current study, *Entamoeba* sp., with “*E. coli* (11%) and *E. histolytica* complex (5.8%)” were detected. *E. coli* was identified in 70% of the studies conducted in Africa whereas *E. histolytica/dispar/moshkovskii*, with a highest estimated prevalence range exceeding 50% ranked as the second most prevalent parasitic infection following giardiasis [1]. While light microscopy remained the prevailing screening method for GIP infection [51], the current study is constrained by the need for molecular methodologies capable of conducting comprehensive analyses of *E. histolytica* complex. Despite *E. coli*, *E. dispar*, and *E. moshkovskii* being classified as a non–pathogenic commensal intestinal protozoa, evidence suggests their proliferation can induce moderate inflammation in the large intestine, leading to abdominal discomfort and diarrhea [50]. This indicates that the population is a reservoir for infections and has inadequate sanitation practices.

The present study unveiled the presence of poly-parasitism in as many as four distinct parasite species, with the highest frequency of co-infection attributed to *Blastocystis* sp. and *Entamoeba* sp. This result aligns with previous investigations that have documented the occurrence of poly-parasitism between *Blastocystis* sp., *Entamoeba* sp., and *G. duodenalis* [32, 44]. This finding offers additional substantiation that the source of the food and water contamination was feces. In rural areas, where various risk factors intersect frequently, mixed parasitic infections are to be anticipated. The research findings indicate that sociodemographic characteristics, such as inadequate personal hygiene among family members, multiple animal species in and around human dwellings, and unsuccessful treatment for previous parasitic infections, contribute to the emergence of such a diverse range of GIP infections. The acquisition of mixed parasitic infections may serve as a basis for evaluating the relationship between morbidity and comorbidity, thereby facilitating the formulation of comprehensive and targeted interventions and measures.

For each decade of increased age, the likelihood of GIP infection decreased by 17%. Age emerged as a critical determinant of risk, as evidenced by the prevalence of GIP infections among preschool and school-aged children in ARE [31, 42] and other countries [43–45]. Risk factors for GIP infections in children included immature immunity, feeding and exploratory behaviors of children under the age of five, uncontrolled hygiene among schoolchildren in the picture of untrimmed fingers, consuming food from street vendors and school canteens, failing to wash hands before eating, after using the restroom, after coming into contact with soil, swimming in surface water, and making hand contact with stray animals [31, 50, 52, 53]. This could also be attributed to participants’ awareness and personal sanitation, which tend to improve as they age.

The odds of GIP infection were approximately four times higher among those who displayed clinical manifestation than those who did not exhibit any symptoms (OR = 4.78, *p* < 0.001). Abdominal pain was the most consistently observed clinical manifestation among the symptomatic patients. Previous studies have similarly identified abdominal pain as the prevailing complaint among patients who have contracted the infection [31, 32, 54]. In contrast to asymptomatic patients, symptomatic patients who reported abdominal pain and diarrhea exhibited a greater prevalence of poly-parasitism [32]. The lack of a conclusive correlation between the presence of symptoms and the development of GIP infections exacerbates the problem. It increases the parasite load when most patients choose not to seek medical intervention. Consequently, the infection’s prevalence remains underestimated because infected individuals continue to infect others while remaining silent carriers [31].

Perianal itching was another significant symptom among symptomatic infected persons (OR = 4.11, *p* < 0.001). Four of the 32 individuals who reported a history of perianal itching were confirmed to have an *E. vermicularis* infection. Concerning neglected procedures in ARE is the diagnosis of *E. vermicularis* infection. It is predominately determined by the history of perianal irritation in children and adults and by the clinical presentation of vulvovaginitis in females. While washing their hands after using the toilet, some patients lament the presence of pin-sized, milk-white adult worms around the anus shed in their palms. Most clinicians provide therapy for the patient and their family concerning one of the preceding items in the patient’s medical history. Without a severe infection, a microscopic examination of the infected person’s feces will not yield a positive result of enterobiasis. This elucidates why the current investigation identified infection in 4 out of 32 cases that reported perianal itching. Indeed, the scotch test not utilized in Egypt to detect *E. vermicularis* may result in the oversight of numerous infections [51].

In this study, animal ownership in or near the participants’ dwellings was a risk factor, with pets being associated with a higher chance of GIP infection (OR = 1.71, *p* = 0.004). Furthermore, exposure to or possessing additional animal species constituted another risk factor (OR = 1.39, *p* = 0.003). Similarly, the frequency of enteric protozoa infection is most significantly and consistently influenced by animal ownership. Individuals who have a prior record of animal ownership and care or reside near animals are at a notably elevated risk of contracting parasitic infection and are exposed to the potential for zoonotic transmission [46]. An Egyptian study [25] documented the presence of *Giardia* sp. assemblages on dogs housed in a shelter and on their caretakers. Another study linked the transmission of GIP among university students to their infected pets [54]. This resulted from the fact that most Egyptians, particularly those who reside in the countryside, keep pets and domestic animals near their residences. This phenomenon was particularly conspicuous in various areas of West Ismailia, where Egyptian accommodations were shared by domestic and farm animals in the absence of separate yards, thereby continuously exposing owners to variable GIP infection.

The potential risk factors associated with individuals’ drinking water are the quality and source of the water. There was a significant association between the purchase of water containers, the consumption of turbid tape water, and the use of water directly from pumps. According to the crude model, individuals who reported consuming turbid water had a 2.25-fold higher chance of GIP infection than those who reported drinking clear water. The outcome of the final adjusted model maintained the significance of this association (OR: 2.53; 95% CI: 1.44–4.47). This could be attributed to an increased likelihood of water contamination occurring during its conveyance from pumps to residences or using contaminated implements for water collection. However, pollution predominantly originates from the discharge of treated drainage into the canal, seepage from garbage, and upstream River Nile discharge. West Ismailia’s rural regions have been particularly severely impacted, with some remote localities lacking access to potable drinking water. This circumstance escalates the potential for contamination throughout the water transit and processing stages [18]. In this regard, numerous protists were discovered in water reservoirs, pumps, and potable water in the governorates of Ismailia, El-Minia, and Fayoum [25, 26, 55].

In the authors’ previous investigations, PPI was standard in West Ismailia rural areas [18]. A prior history of parasitic infection was considerably associated with current GIP infection in the present study (OR = 4.55, *p* < 0.001). The non-compliance of participants with the recommended anti-parasitic medication regimen and incomplete completion of the therapeutic course (including dosage and duration) could account for this outcome. This was confirmed by the present study’s significant association (OR = 3.01, *p* < 0.001) for noncompliance with treatment for a previous parasitic infection among currently afflicted participants. Non-compliance with the therapeutic regimen and the continued presence of risk factors will lead to the persistence of the disease, reinfection with the same parasites, and the perpetuation of its life cycles.

This study identified 18 families infected with multiple members with identical intestinal protists. It has been established that vendors who provide care for children who are afflicted are susceptible to acquiring the infection themselves [52]. Another study documented that contact with family members with gastrointestinal disorders constituted the most significant risk factor for protist infections [56]. Furthermore, standard behaviors within the family, such as neglecting to cleanse hands thoroughly with soap and water before meals and after using the toilet, increase the risk of infection for other family members. A perpetual cycle of GIP infection will ensue unless the entire family is treated simultaneously, emphasizing adhering to hygienic standards and averting risk factors.

Due to the large number of samples collected simultaneously and the absence of technicians conducting microscopic examinations, the current study was constrained by permanent stains. While it was not feasible to apply PCR identification to every detected parasitic infestation in the current study due to the lack of funding grants, PCR identification, and sequencing were performed on the most prevalent parasite (*Blastocystis* sp.) found in the population under investigation [18].

## Conclusion

The current study’s results provide a comprehensive summary of the prevalence of GIP in five distinct localities of West Ismailia, Egypt. Protists most commonly encountered among infected participants were *Blastocystis* sp., *G. duodenalis*, and *Entamoeba* sp. These protists were identified singly or in conjunction with one another or other parasites. Age, residing in a rural area, presenting with abdominal pain, owning multiple species of animals (particularly pets), and lacking access to clean drinking water were all potential risk factors for GIP infection. It is strongly advised that children undergo periodic screening and treatment for GIP infection and that the public be educated about the risks and preventative measures of GIP, with particular emphasis on personal hygiene. The remaining family members should be screened for GIP and treated if one becomes infected. These measures can mitigate the occurrence and risks of GIP in the area under investigation. A more profound comprehension of the transmission mechanisms of these parasites in the studied area necessitates the implementation of additional genotyping research and environmental samples analysis.

### Electronic supplementary material

Below is the link to the electronic supplementary material.

Additional File 1

## Data Availability

No datasets were generated or analysed during the current study.

## References

[1] Ahmed SA, Kotepui M, Masangkay FR, Milanez GD, Karanis P (2023). Gastrointestinal parasites in Africa: a review. Adv Parasitol.

[2] Ahmed SA, Karanis P. *Cryptosporidium* and cryptosporidiosis: the perspective from the Gulf countries. Int J Environ Res Public Health. 2020;17:1–34. 10.3390/ijerph17186824 PMC7558405 32962045

[3] La Hoz RM, Morris MI. Intestinal parasites including *Cryptosporidium*, *Cyclospora*, *Giardia*, and *Microsporidia*, *Entamoeba histolytica*, *Strongyloides*, schistosomiasis, and *Echinococcus*: Guidelines from the American Society of Transplantation Infectious Diseases Community of Practice. Clin Transplant. 2019;33. 10.1111/ctr.13618 31145496

[4] Ahmed SA, Karanis P. An overview of methods/techniques for the detection of *Cryptosporidium* in food samples. Parasitol Res. Springer Verlag; 2018. pp. 629–53. 10.1007/s00436-017-5735-0 29350281

[5] Ahmed SA, Guerrero Flórez M, Karanis P (2018). The impact of water crises and climate changes on the transmission of protozoan parasites in Africa. Pathog Glob Health.

[6] Wu Y, Duffey M, Alex SE, Suarez-Reyes C, Clark EH, Weatherhead JE (2022). The role of helminths in the development of non-communicable diseases. Front Immunol.

[7] Dattani S, Spooner F, Ritchie H, Roser M. Diarrheal diseases - our world in data. Our World in Data; 2023.

[8] Mejia R, Damania A, Jeun R, Bryan PE, Vargas P, Juarez M et al. Impact of intestinal parasites on microbiota and cobalamin gene sequences: a pilot study. Parasit Vectors. 2020;13. 10.1186/s13071-020-04073-7 PMC7168842 32306993

[9] Braseth AL, Elliott DE, Ince MN (2021). Parasitic infections of the gastrointestinal track and liver. Gastroenterol Clin North Am.

[10] GBD Diarrhoeal Diseases Collaboratores (2017). Estimates of global, regional, and national morbidity, mortality, and aetiologies of diarrhoeal diseases: a systematic analysis for the global burden of Disease Study 2015. Lancet Infect Dis.

[11] Fadladdin YAJ, Rahman HU, Kabir M (2022). New record of parasitic infection among school children of Lower Dir Pakistan. Braz J Biol.

[12] Otieno BIA, Matey EJ, Bi X, Tokoro M, Mizuno T, Panikulam A (2023). Intestinal parasitic infections and risk factors for infection in Kenyan children with and without HIV infection. Parasitol Int.

[13] Koop A, Mapped. The 25 poorest countries in the world - visual capitalist. https://www.visualcapitalist.com/mapped-the-25-poorest-countries-in-the-world/

[14] Pickbourn L, Ndikumana L (2019). Does health aid reduce infant and child mortality from diarrhoea in Sub-saharan Africa?. J Dev Stud.

[15] Cabada MM, Morales ML, Lopez M, Reynolds ST, Vilchez EC, Lescano AG, et al. *Hymenolepis nana* impact among children in the highlands of Cusco, Peru: an emerging neglected parasite infection. Am J Trop Med Hyg. 2016;95:1031–6. 10.4269/ajtmh.16-0237 PMC5094212 27672206

[16] Francis L, Kirunda BE, Orach CG (2012). Intestinal helminth infections and nutritional status of children attending primary schools in Wakiso District, Central Uganda. Int J Environ Res Public Health.

[17] Wasihun AG, Teferi M, Negash L, Marugán J, Yemane D, McGuigan KG et al. Intestinal parasitosis, anaemia and risk factors among pre-school children in Tigray region, northern Ethiopia. BMC Infect Dis. 2020;20. 10.1186/s12879-020-05101-8 PMC7251880 32460777

[18] Ahmed SA, El-Mahallawy HS, Mohamed SF, Angelici MC, Hasapis K, Saber T et al. Subtypes and phylogenetic analysis of *Blastocystis* sp. isolates from West Ismailia, Egypt. Sci Reports 2022 12:1. 2022;12:1–12. 10.1038/s41598-022-23360-0 PMC9873628 36351984

[19] Horikoshi Y, Ibrahim UM, Morris SK (2021). School-based approach for parasitic disease control in Japan and Africa. Pediatr Int.

[20] Oyeyemi OT, de Jesus Jeremias W, Grenfell RFQ (2020). Schistosomiasis in Nigeria: gleaning from the past to improve current efforts towards control. One Health.

[21] Ramzy RMR, Al Kubati AS (2020). Progress towards elimination of lymphatic filariasis in the Eastern Mediterranean Region. Int Health.

[22] Gebrezgabiher G, Mekonnen Z, Yewhalaw D, Hailu A (2019). Reaching the last mile: main challenges relating to and recommendations to accelerate onchocerciasis elimination in Africa. Infect Dis Poverty.

[23] Meribo K, Kebede B, Feleke SM, Mengistu B, Mulugeta A, Sileshi M (2017). Review of Ethiopian onchocerciasis elimination programme. Ethiop Med J.

[24] Chiamah O, Ubachukwu P, Anorue C, Ebi S (2019). Urinary schistosomiasis in Ebonyi State, Nigeria from 2006 to 2017. J Vector Borne Dis.

[25] Khalifa RMA, Ahmad AK, Abdel-Hafeez EH, Mosllem FA (2014). Present status of protozoan pathogens causing water-borne disease in northern part of El-Minia Governorate, Egypt. J Egypt Soc Parasitol.

[26] Sakran TF, El-Shahawy GA, Shalaby MA, Sabry HY, Matooq PM, Elmallah AM (2017). Detection rates of waterborne protozoa in water sources from Fayoum Governorate. Parasitologists United J.

[27] Ben Ayed L, Ahmed SAA, Boughattas S, Karanis P. Waterborne *Cryptosporidium* and *Giardia* in resources of MENA: A systematic review and meta-analysis. JWH. 2024; In Press. 10.2166/wh.2024.107 39212283

[28] Rujeni N, Morona D, Ruberanziza E, Mazigo HD (2017). Schistosomiasis and soil-transmitted helminthiasis in Rwanda: an update on their epidemiology and control. Infect Dis Poverty.

[29] Barakat RMR (2013). Epidemiology of schistosomiasis in Egypt: travel through time: review. J Adv Res.

[30] Wikipedia. Ismailia Canal. 2023. https://en.wikipedia.org/wiki/Ismaïlia_Canal

[31] Elmonir W, Elaadli H, Amer A, El-Sharkawy H, Bessat M, Mahmoud SF (2021). Prevalence of intestinal parasitic infections and their associated risk factors among preschool and school children in Egypt. PLoS ONE.

[32] El-Wakil ES, Zalat RS, El-Badry AA (2023). Mapping gut parasitism patterns in a cohort of egyptians. Sci Rep.

[33] Dyab AK, El-Salahy M, Abdelmoneiem H, Mohammed MF (2016). Prevalence and risk factors associated with intestinal parasitic infection among children in Aswan, Egypt. J Bacteriol Parasitol.

[34] Radwan EH, Hassan AAER, Lotfy WM, El-Mawgood AA, Mashaal HM (2019). The prevalence of intestinal parasite infection in El Behara schoolchildren. Int J Limnol.

[35] Ahmed HM, Abu-Sheishaa GA (2022). Intestinal parasitic infection among school children in Dakahlia governorate, Egypt: a cross-sectional study. Egypt Pediatr Association Gaz 2022.

[36] M’bondoukwé NP, Kendjo E, Mawili-Mboumba DP, Koumba Lengongo JV, Offouga Mbouoronde C, Nkoghe D (2018). Prevalence of and risk factors for malaria, filariasis, and intestinal parasites as single infections or co-infections in different settlements of Gabon, Central Africa. Infect Dis Poverty.

[37] Britannica. Ismailia | Suez Canal, Nile Delta, Oases. Encyclopedia Britannica. 2023.

[38] Country coordinate. GPS coordinates of Ismailia, Egypt. 2023. https://www.countrycoordinate.com/city-ismailia-egypt/

[39] Wikipedia. Ismailia Governorate. Wikimedia Foundation, Inc. 2023. https://en.wikipedia.org/wiki/Ismailia_Governorate

[40] Goher ME, Hassan AM, Abdel-Moniem IA, Fahmy AH, El-Sayed SM (2014). Evaluation of surface water quality and heavy metal indices of Ismailia Canal, Nile River, Egypt. Egypt J Aquat Res.

[41] Amer AS, Mohamed WS (2022). Assessment of Ismailia Canal for irrigation purposes by water quality indices. Environ Monit Assess.

[42] Omar M, Abdelal HO (2022). Current status of intestinal parasitosis among patients attending teaching hospitals in Zagazig district, Northeastern Egypt. Parasitol Res.

[43] Candela E, Goizueta C, Periago MV, Muñoz-Antoli C (2021). Prevalence of intestinal parasites and molecular characterization of Giardia Intestinalis, Blastocystis spp. and Entamoeba histolytica in the village of Fortín Mbororé (Puerto Iguazú, Misiones, Argentina). Parasit Vectors.

[44] Tapia-Veloz E, Gozalbo M, Guillén M, Dashti A, Bailo B, Köster PC et al. Prevalence and associated risk factors of intestinal parasites among schoolchildren in Ecuador, with emphasis on the molecular diversity of *Giardia duodenalis*, *Blastocystis* sp. and *Enterocytozoon bieneusi*. PLoS Negl Trop Dis. 2023;17:e0011339. /pmc/articles/PMC10243618/. 10.1371/journal.pntd.0011339 PMC10243618 37224177

[45] Hassan AO, Salih JM, Al-Saeed ATM (2023). Prevalence of intestinal parasites and associated risk factors among rural areas in Duhok province. J Adv Zool.

[46] Sebaa S, Behnke JM, Baroudi D, Hakem A, Abu-Madi MA (2021). Prevalence and risk factors of intestinal protozoan infection among symptomatic and asymptomatic populations in rural and urban areas of southern Algeria. BMC Infect Dis.

[47] Curtale F, Wahab Hassanein YA, El El A, Barduagni P, Savioli L (2003). The school health programme in Behera: an integrated helminth control programme at Governorate level in Egypt. Acta Trop.

[48] Oyegue-Liabagui SL, Ndjangangoye NK, Kouna LC, Lekolo GM, Mounioko F, Kwedi Nolna S (2020). Molecular prevalence of intestinal parasites infections in children with diarrhea in Franceville, Southeast of Gabon. BMC Infect Dis.

[49] El Fatni C, Olmo F, El Fatni H, Romero D, Rosales MJ. First genotyping of *Giardia duodenalis* and prevalence of entero-parasites in children from Tetouan. (Morocco) Parasite. 2014;21:48. https://www.parasite-journal.org/articles/parasite/full_html/2014/01/parasite140064/parasite140064.html 10.1051/parasite/2014049 PMC4176428 25259605

[50] Biwott P, Wanjala G, Ngeiywa M. Prevalence of gastrointestinal parasitic infections among food handlers in Eldoret municipality, Kenya. J Biol Agric Healthc. 2014.

[51] Ahmed SA, Mohamed SF, Fouad AM, Karanis P (2022). Gastrointestinal parasites diagnoses at the primary health care units: a comparative analysis of diagnostic abilities of parasitology staff technicians versus medical parasitologists in Ismailia, Egypt. Trans R Soc Trop Med Hyg.

[52] Idowu OA, Rowland SA (2006). Oral fecal parasites and personal hygiene of food handlers in Abeokuta, Nigeria. Afr Health Sci.

[53] Fauziah N, Aviani JK, Agrianfanny YN, Fatimah SN. Intestinal parasitic infection and nutritional status in children under five years old: a systematic review. Trop Med Infect Dis. 2022;7:371. 10.3390/tropicalmed7110371 pmc/articles/PMC9697828/. 10.3390/tropicalmed7110371 PMC9697828 36422922

[54] Potes-Morales C, del Crespo-Ortiz P (2023). Molecular diagnosis of intestinal protozoa in young adults and their pets in Colombia, South America. PLoS ONE.

[55] Abd El-Latif NF, El-Taweel HA, Gaballah A, Salem AI, Abd El-Malek AHM. Molecular characterization of *Giardia intestinalis* detected in humans and water samples in Egypt. Acta Parasitol. 2020;65:482–9. https://pubmed.ncbi.nlm.nih.gov/32124205/10.2478/s11686-020-00176-4 32124205

[56] Osman M, El Safadi D, Cian A, Benamrouz S, Nourrisson C, Poirier P et al. Prevalence and risk factors for intestinal protozoan infections with *Cryptosporidium*, *Giardia*, *Blastocystis* and *Dientamoeba* among schoolchildren in Tripoli, Lebanon. PLoS Negl Trop Dis. 2016;10. 10.1371/journal.pntd.0004496 PMC4790957 26974335

