# Supplementary material for: Gastrointestinal parasitic infections: Prevalence and risk factors in West Ismailia, Arab Republic of Egypt
Source: Gut Pathog. 2024 Jun 19;16:29. doi: 10.1186/s13099-024-00622-y (PMC11186246; doi:10.1186/s13099-024-00622-y)
Supplement: Supplementary file 1 — Additional File 1 [file 13099_2024_622_MOESM1_ESM.docx]

**Table S1:** Zoonotic exposure characteristics of West Ismailia population (520 individuals).

| Variable | Category | Number | (%) |
| --- | --- | --- | --- |
| Owing animals | Yes | 474 | 91.2 |
|  | No | 46 | 8.85 |
| Type of animal (if any) | Poultry | 102 | 21.5 |
|  | Pet | 15 | 3.2 |
|  | Livestock | 11 | 2.3 |
|  | Poultry and pet | 211 | 44.5 |
|  | Poultry and livestock | 38 | 8.0 |
|  | Livestock and pet | 3 | 0.6 |
|  | Poultry, pet, and livestock | 94 | 19.8 |
| Direct contact with animal (if any) | Yes | 362 | 76.4 |
|  | No | 112 | 23.6 |
| Type of farm ground (if any) | Sand | 347 | 73.2 |
|  | Cement | 119 | 25.1 |
|  | Mixed | 8 | 1.7 |

**Table S2:** Water facility characteristics of West Ismailia population (520 individuals).

| Variable | Category | Number | % |
| --- | --- | --- | --- |
| Have access to potable water | Yes | 517 | 99.4 |
|  | No | 3 | 0.57 |
| Description of water (if any) | Clear | 327 | 63.3 |
|  | Turbid | 141 | 27.3 |
|  | Yellow | 49 | 9.5 |
| Type of water supply for human consumption | Tap | 370 | 71.2 |
|  | Buying containers | 87 | 16.7 |
|  | Pump | 31 | 6.0 |
|  | Tap with filter | 26 | 5.0 |
|  | Tanks | 6 | 1.2 |
| Type of water source for animals (if any) | Pump | 106 | 22.4 |
|  | Tap | 331 | 69.8 |
|  | Canal | 24 | 5.1 |
|  | Ground water | 11 | 2.3 |
|  | Tanks | 2 | 0.4 |

**Table S3:** Symptomatology status of West Ismailia population (520 individuals).

| Variable |  | Category | Number | % |
| --- | --- | --- | --- | --- |
| Manifestation of symptoms | | Symptomatic | 370 | 71.1 |
|  |  | Asymptomatic | 150 | 28.9 |
| Symptom associated with symptomatic patients* | GIT symptoms | Abdominal pain | 320 | 61.5 |
|  |  | Diarrhoea | 154 | 29.6 |
|  |  | Vomiting | 49 | 9.5 |
|  |  | Bloody stool | 10 | 1.5 |
|  | Non-GIT symptoms | Fever | 59 | 11.4 |
|  |  | Dehydration | 19 | 3.7 |

GIT: Gastrointestinal tract.

*The sum exceeds 100% as participants could report multiple symptoms. 149 individuals had more than one symptom: 2 symptoms (96), 3 symptoms (29), 4 symptoms (9), 5 symptoms (15).

**Table S4:** Prior parasitic infection (PPI) phenotypes and treatment receipt.

| Variable | Category | Number | % |
| --- | --- | --- | --- |
| Have PPI | Yes | 265 | 51 |
|  | No | 255 | 49 |
| Species of parasites in PPI (if any) | *Entamoeba* sp. (*E. coli* or *E. histolytica complex*) | 38 | 14.3 |
|  | *E. vermicularis* | 25 | 9.4 |
|  | *H. nana* | 15 | 5.7 |
|  | *Schistosoma* sp. | 12 | 4.5 |
|  | *Taenia* sp. | 2 | 0.8 |
|  | *Ascaris* sp. | 1 | 0.4 |
|  | Unknown GIP infection | 172 | 64.9 |
| Treatment receipt in PPI | Yes | 103 | 38.9 |
|  | No | 162 | 61.1 |

*E. coli*: *Entamoeba coli*; *E.* *histolytica* complex: *Entamoeba histolytica*/*dispar*/*moshkovskii*; *E. vermicularis*: *Enterobius vermicularis*; *H. nana*: *Hymenolepis nana*.

**Table S5:** Factors associated with *Entamoeba* sp. infection in West Ismailia population (univariable and multivariable analysis).

| Factor | Categories | cOR | p-value | 95% CI | | aOR | p-value | | | 95% CI | | |
| --- | --- | --- | --- | --- | --- | --- | --- | --- | --- | --- | --- | --- |
| Gender^a^ | Male | 0.91 | 0.691 | 0.56 | 1.47 | - | | | | | | |
| Age | (per 10 years increase) | 0.91 | 0.239 | 0.78 | 1.06 | - | | | | | | |
| Age groups | 0-4 | 1.18 | 0.719 | 0.49 | 2.85 | - | | | | | | |
|  | 5-9 | 1.25 | 0.572 | 0.58 | 2.69 | - | | | | | | |
|  | 10-14 | 1.68 | 0.184 | 0.78 | 3.59 | - | | | | | | |
|  | 15-34 | 0.99 | 0.975 | 0.44 | 2.21 | - | | | | | | |
| Residence^b^ | Abu-Swayer | 2.07 | 0.115 | 0.84 | 5.14 | - | | | | | | |
|  | El-Kassassin | 1.20 | 0.643 | 0.55 | 2.61 |  |  |  |  |  |  |  |
|  | El-Mahsama | 2.02 | 0.029* | 1.08 | 3.78 |  |  |  |  |  |  |  |
|  | Ismailia | - | | | |  |  |  |  |  |  |  |
| Symptoms^c^ | Any | 3.09 | 0.001* | 1.59 | 6.00 | 2.57 | 0.009* | | | 1.26 | 5.22 | |
|  | Diarrhea | 0.90 | 0.679 | 0.53 | 1.51 | - | | | | | | |
|  | Bloody stools | 1.32 | 0.726 | 0.28 | 6.35 | - | | | | | | |
|  | Vomiting | 1.03 | 0.942 | 0.46 | 2.29 | - | | | | | | |
|  | Fever | 0.94 | 0.875 | 0.44 | 2.00 | - | | | | | | |
|  | Abdominal pain | 2.38 | 0.002* | 1.38 | 4.11 | - | | | | | | |
|  | Dehydration | 0.61 | 0.514 | 0.14 | 2.69 | - | | | | | | |
|  | Perianal itching | 2.19 | 0.058 | 0.98 | 4.92 | 2.43 | | 0.044* | 1.02 | | | 5.78 |
| Zoonotic exposure | Pet^c^ | 1.16 | 0.547 | 0.71 | 1.90 | - | | | | | | |
|  | Poultry^c^ | 1.12 | 0.741 | 0.57 | 2.23 | - | | | | | | |
|  | Livestock^c^ | 1.13 | 0.651 | 0.67 | 1.88 | - | | | | | | |
|  | No. of animals’ species (continuous) | 1.11 | 0.472 | 0.84 | 1.47 | - | | | | | | |
|  | Direct contact with animals^c^ | 1.82 | 0.073 | 0.95 | 3.52 | 2.11 | 0.033* | | | 1.06 | 4.20 | |
| Water description^d^ | Turbid | 1.57 | 0.080 | 0.95 | 2.61 | 1.72 | 0.054 | | | 0.99 | 3.00 | |
|  | Yellow | 0.66 | 0.404 | 0.25 | 1.75 | 0.49 | 0.163 | | | 0.18 | 1.34 | |
| Water supply to human^e^ | Buying containers | 0.60 | 0.156 | 0.29 | 1.22 | - | | | | | | |
|  | Pump | 0.68 | 0.489 | 0.23 | 2.02 |  |  |  |  |  |  |  |
|  | Tank | - | | | |  |  |  |  |  |  |  |
|  | Tap | 0.60 | 0.418 | 0.18 | 2.06 |  |  |  |  |  |  |  |
| Water source to animal^f^ | Canal | 0.67 | 0.531 | 0.19 | 2.33 | - | | | | | | |
|  | Ground water | 0.47 | 0.477 | 0.06 | 3.75 |  |  |  |  |  |  |  |
|  | Pump | 0.78 | 0.419 | 0.42 | 1.44 |  |  |  |  |  |  |  |
| PPI | PPI^c^ | 2.75 | <0.001* | 1.65 | 4.57 | 2.17 | 0.007* | | | 1.24 | 3.81 | |
|  | TTT of PPI^g^ | 1.59 | 0.144 | 0.85 | 2.95 | - | | | | | | |

Superscripts are variables that have a reference category; ^a^ Female; ^b^ El-Talelkbeer, ^c^ Yes; ^d^ Clear; ^e^ Tap with filter; ^f^ Tap (i.e. without filter); ^g^ No; *p-value <0.05; GIP: Gastrointestinal parasites; cOR: Crude odds ratio; aOR: Adjusted odds ratio; CI: Confidence interval; PPI: Prior parasitic infection; TTT: Treatment.

**Table S6:** Factors associated with *Blastocystis* sp. infection in West Ismailia population (univariable and multivariable analysis).

| Factor | Categories | cOR | p-value | 95% CI | | aOR | | p-value | | 95% CI | |
| --- | --- | --- | --- | --- | --- | --- | --- | --- | --- | --- | --- |
| Gender^a^ | Male | 0.98 | 0.924 | 0.60 | 1.59 | - | | | | | |
| Age | (per 10 years increase) | 0.94 | 0.508 | 0.98 | 1.01 | - | | | | | |
|  | 0-4 | 0.78 | 0.620 | 0.28 | 2.12 | - | | | | | |
| Age group | 5-9 | 1.15 | 0.734 | 0.52 | 2.57 | - | | | | | |
|  | 10-14 | 1.96 | 0.090 | 0.90 | 4.25 | - | | | | | |
|  | 15-34 | 1.42 | 0.388 | 0.64 | 3.13 | - | | | | | |
| Residence^b^ | Abu-Swayer | 0.88 | 0.874 | 0.18 | 4.39 | 0.53 | | 0.472 | 0.09 | | 3.02 |
|  | El-Kassassin | 1.68 | 0.322 | 0.60 | 4.66 | 0.86 | | 0.823 | 0.24 | | 3.10 |
|  | El-Mahsama | 7.01 | <0.001* | 3.10 | 15.84 | 3.05 | | 0.049* | 1.00 | | 9.27 |
|  | Ismailia | - | | | | - | | | | | |
| Symptoms^c^ | Any | 5.07 | <0.001* | 2.28 | 11.30 | 2.14 | | 0.117 | | 0.83 | 5.55 |
|  | Diarrhea | 0.82 | 0.474 | 0.48 | 1.41 | - | | | | | |
|  | Bloody stools | - | | | | - | | | | | |
|  | Vomiting | 0.60 | 0.296 | 0.23 | 1.56 | - | | | | | |
|  | Fever | 0.59 | 0.243 | 0.25 | 1.43 | - | | | | | |
|  | Abdominal pain | 5.32 | <0.001* | 2.67 | 10.60 | - | | | | | |
|  | Dehydration | 0.30 | 0.240 | 0.04 | 2.26 | - | | | | | |
|  | Perianal itching | 1.29 | 0.587 | 0.51 | 3.25 |  | | | | | |
| Zoonotic exposure | Pet^c^ | 2.80 | 0.001* | 1.57 | 4.99 | - | | | | | |
|  | Poultry^c^ | 7.76 | 0.005* | 1.86 | 32.28 | - | | | | | |
|  | Livestock^c^ | 0.83 | 0.506 | 0.48 | 1.43 | - | | | | | |
|  | No. of animals’ species (continuous) | 1.60 | 0.002* | 1.18 | 2.17 | 1.57 | | 0.050 | | 1.00 | 2.47 |
|  | Direct contact with animals^c^ | 1.13 | 0.677 | 0.63 | 2.03 | - | | | | | |
| Water description^d^ | Turbid | 4.46 | <0.001* | 2.69 | 7.41 | 1.79 | | 0.175 | | 0.77 | 4.13 |
|  | Yellow | 0.39 | 0.209 | 0.09 | 1.69 | 0.28 | | 0.102 | | 0.06 | 1.29 |
| Water supply to human^e^ | Buying containers | 0.31 | 0.009 | 0.13 | 0.74 | - | | | | | |
|  | Pump | 0.14 | 0.055 | 0.02 | 1.05 | - | | | | | |
|  | Tank | - | | | | - | | | | | |
|  | Tap | 0.35 | 0.161 | 0.08 | 1.52 | - | | | | | |
| Water source to animal^f^ | Canal | 0.35 | 0.157 | 0.08 | 1.50 | - | | | | | |
|  | Ground water | - | | | | - | | | | | |
|  | Pump | 0.27 | 0.001* | 0.12 | 0.60 | - | | | | | |
| PPI | PPI^c^ | 52.56 | <0.001* | 12.75 | 216.62 | 24.56 | | <0.001* | | 4.81 | 125.32 |
|  | TTT of PPI^g^ | 4.59 | <0.001* | 2.37 | 8.89 | 2.04 | 0.129 | | | 0.81 | 5.12 |

Superscripts are variables that have a reference category; ^a^ Female; ^b^ El-Talelkbeer, ^c^ Yes; ^d^ Clear; ^e^ Tap with filter; ^f^ Tap (i.e. without filter); ^g^ No; *p-value <0.05; GIP: Gastrointestinal parasites; cOR: Crude odds ratio; aOR: Adjusted odds ratio; CI: Confidence interval; PPI: Prior parasitic infection; TTT: Treatment.

**Table S7:** Factors associated with *Giardia duodenalis* infection in West Ismailia population (univariable and multivariable analysis).

| Factor | Categories | cOR | p-value | 95% CI | | aOR | p-value | 95% CI | |
| --- | --- | --- | --- | --- | --- | --- | --- | --- | --- |
| Gender^a^ | Male | 1.27 | 0.374 | 0.75 | 2.15 | - | | | |
| Age | (per 10 years increase) | 0.56 | 0.001* | 0.41 | 0.74 | - | | | |
| Age group | 0-4 | 10.47 | <0.001* | 2.96 | 37.13 | 7.63 | 0.003* | 1.97 | 29.53 |
|  | 5-9 | 5.57 | 0.007* | 1.61 | 19.31 | 4.70 | 0.02* | 1.27 | 17.37 |
|  | 10-14 | 3.80 | 0.042* | 1.05 | 13.79 | 3.87 | 0.048* | 1.01 | 14.78 |
|  | 15-34 | 2.05 | 0.300 | 0.53 | 7.95 | 1.74 | 0.447 | 0.42 | 7.21 |
| Residence^b^ | Abu-Swayer | 1.49 | 0.446 | 0.53 | 4.20 | - | | | |
|  | El-Kassassin | 1.18 | 0.692 | 0.52 | 2.71 | - | | | |
|  | El-Mahsama | 1.57 | 0.196 | 0.79 | 3.11 | - | | | |
|  | Ismailia | 1.58 | 0.684 | 0.18 | 14.13 | - | | | |
| Symptoms^c^ | Any | 2.14 | 0.029* | 1.08 | 4.21 | 2.49 | 0.018* | 1.17 | 5.28 |
|  | Diarrhea | 1.39 | 0.238 | 0.80 | 2.41 | - | | | |
|  | Bloody stools | 0.79 | 0.823 | 0.10 | 6.33 | - | | | |
|  | Vomiting | 0.44 | 0.177 | 0.13 | 1.45 | 0.18 | 0.018* | 0.04 | 0.74 |
|  | Fever | 0.95 | 0.912 | 0.41 | 2.20 | - | | | |
|  | Abdominal pain | 1.13 | 0.658 | 0.66 | 1.95 | - | | | |
|  | Dehydration | 1.35 | 0.639 | 0.38 | 4.78 | - | | | |
|  | Perianal itching | 3.08 | 0.007* | 1.36 | 7.00 | - | | | |
| Zoonotic exposure | Pet^c^ | 1.29 | 0.373 | 0.74 | 2.24 | - | | | |
|  | Poultry^c^ | 1.03 | 0.930 | 0.49 | 2.19 | - | | | |
|  | Livestock^c^ | 2.40 | 0.001* | 1.41 | 4.10 | 3.23 | 0.001* | 1.62 | 6.44 |
|  | No. of animals’ species (continuous) | 1.46 | 0.024* | 1.05 | 2.03 | 1.62 | 0.015* | 1.10 | 2.38 |
|  | Direct contact with animals^c^ | 0.63 | 0.119 | 0.35 | 1.13 | 0.52 | 0.069 | 0.26 | 1.05 |
| Water description^d^ | Turbid | 1.15 | 0.655 | 0.63 | 2.09 | - | | | |
|  | Yellow | 1.09 | 0.849 | 0.44 | 2.75 | - | | | |
| Water supply to human^e^ | Buying containers | 0.70 | 0.368 | 0.32 | 1.53 | 12.06 | 0.035* | 1.20 | 121.51 |
|  | Pump | 0.47 | 0.318 | 0.11 | 2.05 | 4.53 | 0.264 | 0.32 | 64.21 |
|  | Tank | 6.87 | 0.02* | 1.35 | 35.05 | 632.47 | 0.001* | 13.62 | 29377.50 |
|  | Tap | 1.25 | 0.694 | 0.41 | 3.79 | 1.36 | 0.630 | 0.39 | 4.79 |
| Water source to animal^f^ | Canal | 0.89 | 0.848 | 0.25 | 3.09 | 0.13 | 0.149 | 0.01 | 2.07 |
|  | Ground water | 10.84 | 0.001* | 3.05 | 38.51 | 2.33 | 0.397 | 0.33 | 16.42 |
|  | Pump | 0.24 | 0.008* | 0.09 | 0.69 | 0.02 | 0.002* | 0.00 | 0.25 |
| PPI | PPI^c^ | 3.08 | 0.007* | 1.36 | 7.00 | - | | | |
|  | TTT of PPI^g^ | 1.10 | 0.724 | 0.65 | 1.86 | - | | | |

Superscripts are variables that have a reference category; ^a^ Female; ^b^ El-Talelkbeer, ^c^ Yes; ^d^ Clear; ^e^ Tap with filter; ^f^ Tap (i.e. without filter); ^g^ No; *p-value <0.05; GIP: Gastrointestinal parasites; cOR: Crude odds ratio; aOR: Adjusted odds ratio; CI: Confidence interval; PPI: Prior parasitic infection; TTT: Treatment.
